# 4-(1*H*-2,3-Dihydronaphtho­[1,8-*de*][1,3,2]di­aza­borinin-2-yl)-1-ethylpyridin-1-ium iodide monohydrate

**DOI:** 10.1107/S2414314624003699

**Published:** 2024-04-26

**Authors:** Shu Hashimoto, Shintaro Miki, Tsunehisa Okuno

**Affiliations:** a Department of Systems Engineering, Wakayama University, Sakaedani, Wakayama, 640-8510, Japan; University of Aberdeen, United Kingdom

**Keywords:** crystal structure, hydrate polymorph, pyridinium ion, Bdan

## Abstract

The cation of the title hydrated salt, C_17_H_17_BN_3_^+^·I^−^·H_2_O, is a di­aza­borinane featuring substitution at the 1, 2, and 3 positions in the nitro­gen–boron six-membered heterocycle. The cation is approximately planar with a dihedral angle between the pyridyl ring and the di­aza­borinane ring system of 5.40 (5)°. In the crystal, the cations stack along [100] in an alternating head-to-tail manner, while the iodide ion and water mol­ecule form one-dimensional hydrogen-bonded chains beside the cation stack. The cation stacks and I^−^–water chains are crosslinked by N—H⋯I and N—H⋯O hydrogen bonds.

## Abstract 2

The cation of the title hydrated salt is a di­aza­borinane featuring substitution at 1, 2, and 3 positions in the nitro­gen–boron six-membered heterocycle. In the crystal, the cations stack along [100] in an alternating head-to-tail manner, while the iodide ion and water mol­ecule form one-dimensional hydrogen-bonded chains beside the cation stack. The cation stacks and I^−^–water chains are crosslinked by N—H⋯I and N—H⋯O hydrogen bonds.

## Graphical abstract

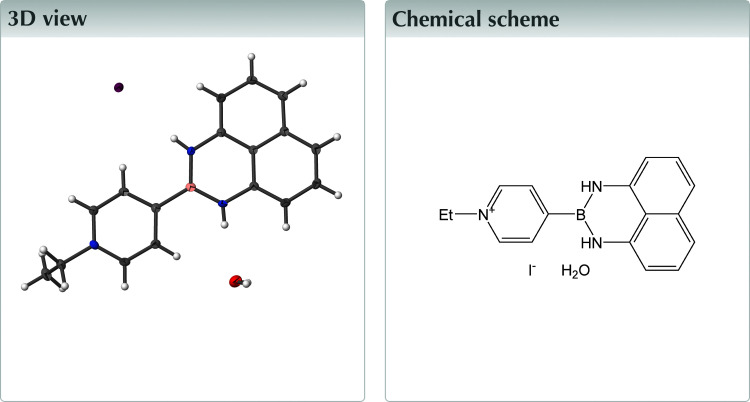

## Structure description

**Figure 1:**
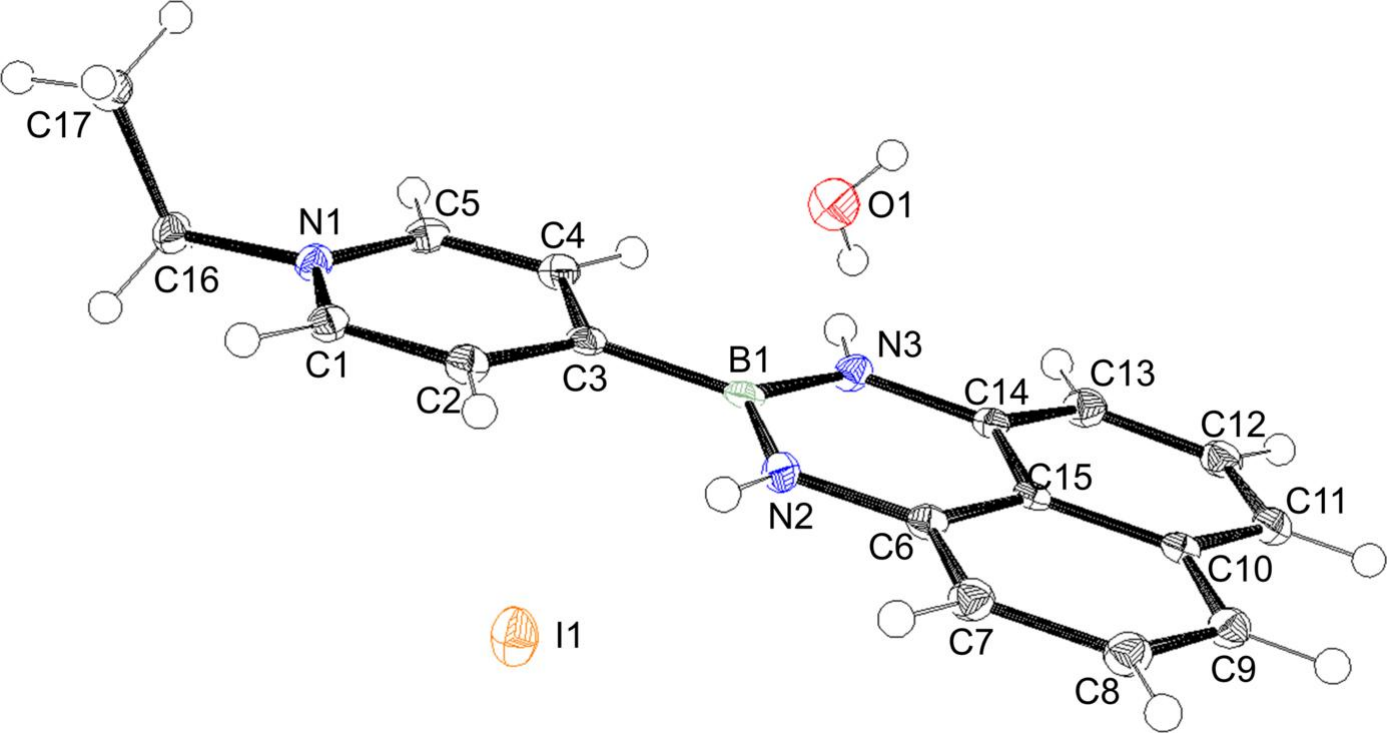
The asymmetric unit of the title compound with displacement ellipsoids drawn at the 50% probability level.

The title compound, C_17_H_17_BN_3_^+^·I^−^·H_2_O, is a hydrated di­aza­borinane derivative featuring substitution at the 1, 2, and 3 positions in the nitro­gen–boron six-membered heterocycle (Fig. 1 ▸). Di­aza­borinanes are found to stabilize organic radicals (LaPorte *et al.*, 2023 ▸). Recently we reported of the anhydrous polymorph of the title compound (Hashimoto & Okuno, 2024 ▸).

In the hydrated polymorph, the organic unit is almost planar with a dihedral angle between the N1/C1–C5 pyridyl ring and N2/N3/C6–C15/B1 ring system of 5.40 (5)°. The structure is similar to those of the anhydrous polymorph and other di­aza­borinanes (Akerman *et al.*, 2011 ▸; Hashimoto & Okuno, 2024 ▸; Slabber *et al.*, 2011 ▸).

**Figure 2:**
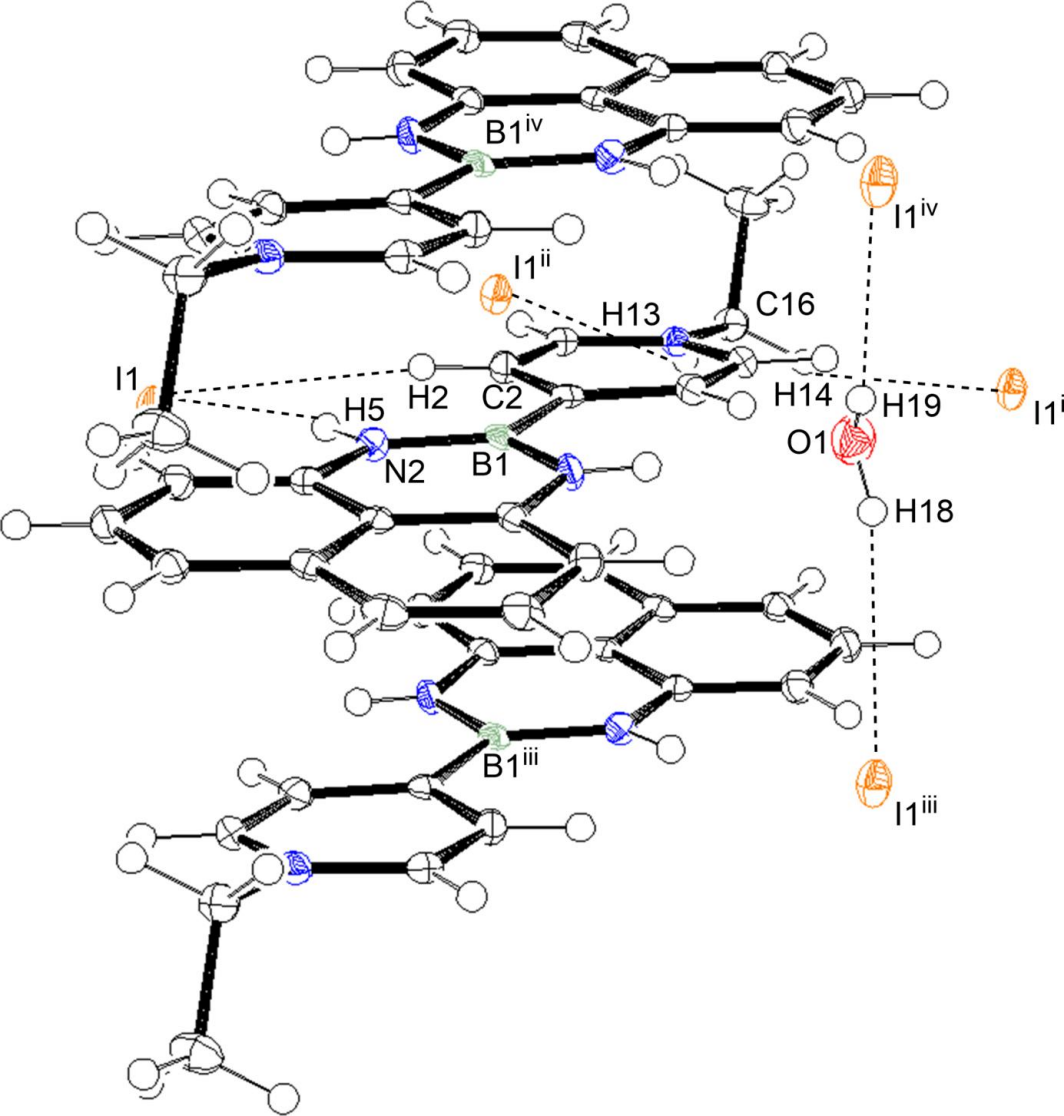
Inter­molecular inter­actions of the title compound. [symmetry codes: (i) *x*, *y* − 1, *z*; (ii) *x*, −*y* + 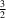 , *z* − 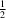 ; (iii) −*x* + 2, −*y* + 2, −*z* + 1; (iv) −*x* + 1, −*y* + 2, −*z* + 1].

In the crystal, the organic cations stack in an alternating head-to-tail manner along the *a* axis as shown in Fig. 2 ▸, where the B1⋯·B1^iv^ and B1⋯·B1^iii^ distances are 3.395 (6) and 3.436 (6) Å, respectively [symmetry codes: (iv) −*x* + 1, −*y* + 2, −*z* + 1; (iii) −*x* + 2, −*y* + 2, −*z* + 1]. The iodide ion accepts three C—H⋯I contacts from adjacent cations and two O—H⋯I links from the water mol­ecules. The iodide anions and water mol­ecules form a one-dimensional hydrogen-bonded chain beside the alternating cation stack and the stacks and hydrogen-bond chains are crosslinked by N—H⋯O and N—H⋯I links. The geometry of the hydrogen bonds is summarized in Table 1 ▸. The contamination of water in aceto­nitrile is thought to give the hydrated polymorph. Selective formation of the hydrated polymorph has not yet been achieved.

## Synthesis and crystallization

Single crystals in the form of pale-yellow blocks of sufficient quality were obtained by recrystallization of 1-ethyl-4-(1*H*-naphtho­[1,8-*de*] [1,3,2]di­aza­borinin-2(3*H*)-yl)pyridin-1-ium iodide (Hashimoto & Okuno, 2024 ▸) from aceto­nitrile solution, which was apparently contaminated with water.

## Refinement

Experimental details and crystal data are summarized in Table 2 ▸.

## Supplementary Material

Crystal structure: contains datablock(s) I. DOI: 10.1107/S2414314624003699/hb4467sup1.cif

Structure factors: contains datablock(s) I. DOI: 10.1107/S2414314624003699/hb4467Isup2.hkl

Supporting information file. DOI: 10.1107/S2414314624003699/hb4467Isup3.cml

CCDC reference: 2350218

Additional supporting information:  crystallographic information; 3D view; checkCIF report

## Figures and Tables

**Table 1:** Hydrogen-bond geometry (Å, °)

*D*—H⋯*A*	*D*—H	H⋯*A*	*D*⋯*A*	*D*—H⋯*A*
O1—H18⋯I1^i^	0.80 (4)	2.87 (4)	3.643 (3)	162 (4)
O1—H19⋯I1^ii^	0.77 (4)	3.04 (4)	3.793 (3)	167 (4)
N2—H5⋯I1	0.83 (3)	2.95 (3)	3.764 (2)	171 (2)
N3—H12⋯O1	0.82 (3)	2.23 (3)	3.046 (3)	172 (3)
C2—H2⋯I1	0.95	3.14	4.081 (2)	171
C16—H14⋯I1^iii^	0.99	2.98	3.840 (3)	145
C16—H13⋯I1^iv^	0.99	3.15	3.946 (3)	138

Symmetry codes: (i) 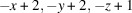 ; (ii) 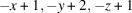 ; (iii) 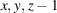 ; (iv) 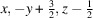 .

**Table 2:** Experimental details

Crystal data	
Chemical formula	C_17_H_17_BN_3_^+^·I^−^·H_2_O
*M*_r_	419.06
Crystal system, space group	Monoclinic, *P*2_1_/*c*
Temperature (K)	93
*a*, *b*, *c* (Å)	6.746 (2), 23.041 (7), 10.939 (3)
β (°)	97.616 (5)
*V* (Å^3^)	1685.4 (9)
*Z*	4
Radiation type	Mo *K*α
μ (mm^−1^)	1.91
Crystal size (mm)	0.18 × 0.18 × 0.10

Data collection	
Diffractometer	Saturn724+
Absorption correction	Numerical (*NUMABS*; Rigaku, 1999 ▸)
*T*_min_, *T*_max_	0.879, 0.900
No. of measured, independent and observed [*I* > 2σ(*I*)] reflections	13511, 3846, 3463
*R*_int_	0.030
(sin θ/λ)_max_ (Å^−1^)	0.649

Refinement	
*R*[*F* ^2^ > 2σ(*F* ^2^)], *wR*(*F* ^2^), *S*	0.026, 0.056, 1.04
No. of reflections	3846
No. of parameters	224
H-atom treatment	H atoms treated by a mixture of independent and constrained refinement
Δρ_max_, Δρ_min_ (e Å^−3^)	0.92, −0.40

Computer programs: *CrystalClear* (Rigaku, 2008 ▸), *SHELXT2014/4* (Sheldrick, 2015*a* ▸), *SHELXL2014/7* (Sheldrick, 2015*b* ▸) and *OLEX2* (Dolomanov *et al.*, 2009 ▸).

## full crystallographic data

### Crystal data

**Table:**

C_17_H_17_BN_3_^+^·I^−^·H_2_O	*F*(000) = 832
*M**_r_* = 419.06	*D*_x_ = 1.652 Mg m^−^^3^
Monoclinic, *P*2_1_/*c*	Mo *K*α radiation, λ = 0.71075 Å
*a* = 6.746 (2) Å	Cell parameters from 6255 reflections
*b* = 23.041 (7) Å	θ = 1.8–30.9°
*c* = 10.939 (3) Å	µ = 1.91 mm^−^^1^
β = 97.616 (5)°	*T* = 93 K
*V* = 1685.4 (9) Å^3^	Block, pale yellow
*Z* = 4	0.18 × 0.18 × 0.10 mm

### Data collection

**Table:**

Saturn724+ diffractometer	3463 reflections with *I* > 2σ(*I*)
Detector resolution: 28.445 pixels mm^-1^	*R*_int_ = 0.030
ω scans	θ_max_ = 27.5°, θ_min_ = 3.2°
Absorption correction: numerical (*NUMABS*; Rigaku, 1999)	*h* = −6→8
*T*_min_ = 0.879, *T*_max_ = 0.900	*k* = −22→29
13511 measured reflections	*l* = −14→14
3846 independent reflections	

### Refinement

**Table:**

Refinement on *F*^2^	0 restraints
Least-squares matrix: full	Hydrogen site location: mixed
*R*[*F*^2^ > 2σ(*F*^2^)] = 0.026	H atoms treated by a mixture of independent and constrained refinement
*wR*(*F*^2^) = 0.056	*w* = 1/[σ^2^(*F*_o_^2^) + (0.0201*P*)^2^ + 1.9341*P*] where *P* = (*F*_o_^2^ + 2*F*_c_^2^)/3
*S* = 1.04	(Δ/σ)_max_ = 0.002
3846 reflections	Δρ_max_ = 0.92 e Å^−^^3^
224 parameters	Δρ_min_ = −0.40 e Å^−^^3^

### Special details

**Table:**

Geometry. All esds (except the esd in the dihedral angle between two l.s. planes) are estimated using the full covariance matrix. The cell esds are taken into account individually in the estimation of esds in distances, angles and torsion angles; correlations between esds in cell parameters are only used when they are defined by crystal symmetry. An approximate (isotropic) treatment of cell esds is used for estimating esds involving l.s. planes.
Refinement. The positions of the N-bound and O-bound H atoms were obtained from difference Fourier maps and were refined isotropically. The C-bound H atoms were placed at ideal positions and were refined as riding on their parent C atoms. *U*_iso_(H) values of the H atoms were set at 1.2*U*_eq_(carrier) or 1.5*U*_eq_(methyl carrier).

### Fractional atomic coordinates and isotropic or equivalent isotropic displacement parameters (Å^2^)

**Table:**

	*x*	*y*	*z*	*U*_iso_*/*U*_eq_	
I1	0.89530 (2)	0.83028 (2)	0.76189 (2)	0.01877 (6)	
O1	0.6094 (4)	1.10388 (9)	0.20283 (18)	0.0265 (4)	
N1	0.7490 (3)	0.86217 (8)	0.19278 (17)	0.0134 (4)	
N2	0.7739 (3)	0.97508 (8)	0.61095 (18)	0.0129 (4)	
N3	0.7269 (3)	1.05026 (8)	0.45657 (18)	0.0134 (4)	
C9	0.7933 (3)	1.09991 (10)	0.8927 (2)	0.0151 (5)	
H8	0.8004	1.1279	0.9569	0.018*	
C3	0.7379 (3)	0.94374 (10)	0.3791 (2)	0.0126 (4)	
C14	0.7397 (3)	1.09351 (10)	0.5472 (2)	0.0125 (4)	
C12	0.7480 (3)	1.19358 (10)	0.6120 (2)	0.0164 (5)	
H10	0.7463	1.2336	0.5911	0.020*	
C5	0.7167 (3)	0.91855 (10)	0.1633 (2)	0.0150 (5)	
H4	0.6979	0.9300	0.0791	0.018*	
C7	0.7968 (3)	0.99955 (10)	0.8283 (2)	0.0152 (5)	
H6	0.8050	0.9596	0.8500	0.018*	
C11	0.7647 (3)	1.17771 (10)	0.7336 (2)	0.0165 (5)	
H9	0.7718	1.2068	0.7956	0.020*	
C1	0.7676 (3)	0.84457 (10)	0.3109 (2)	0.0139 (4)	
H1	0.7844	0.8045	0.3298	0.017*	
C13	0.7334 (3)	1.15171 (10)	0.5175 (2)	0.0159 (5)	
H11	0.7192	1.1635	0.4336	0.019*	
C4	0.7108 (3)	0.95958 (10)	0.2540 (2)	0.0148 (5)	
H3	0.6880	0.9991	0.2318	0.018*	
C8	0.8044 (3)	1.04216 (10)	0.9213 (2)	0.0163 (5)	
H7	0.8174	1.0305	1.0053	0.020*	
C17	0.5808 (4)	0.78797 (11)	0.0480 (2)	0.0214 (5)	
H16	0.6051	0.7605	−0.0168	0.026*	
H15	0.5339	0.7668	0.1165	0.026*	
H17	0.4789	0.8161	0.0145	0.026*	
C15	0.7622 (3)	1.07573 (9)	0.6732 (2)	0.0117 (4)	
C6	0.7776 (3)	1.01577 (10)	0.7059 (2)	0.0126 (4)	
C10	0.7714 (3)	1.11839 (10)	0.7680 (2)	0.0133 (4)	
C2	0.7624 (3)	0.88432 (10)	0.4052 (2)	0.0144 (4)	
H2	0.7756	0.8713	0.4883	0.017*	
C16	0.7726 (3)	0.81952 (10)	0.0940 (2)	0.0161 (5)	
H13	0.8756	0.7907	0.1259	0.019*	
H14	0.8206	0.8400	0.0240	0.019*	
B1	0.7452 (4)	0.99074 (11)	0.4849 (2)	0.0127 (5)	
H12	0.704 (4)	1.0625 (12)	0.386 (3)	0.024 (8)*	
H5	0.787 (4)	0.9418 (13)	0.639 (3)	0.022 (8)*	
H18	0.704 (6)	1.1246 (18)	0.201 (4)	0.058 (13)*	
H19	0.517 (6)	1.1225 (17)	0.212 (3)	0.049 (12)*	

### Atomic displacement parameters (Å^2^)

**Table:**

	*U*^11^	*U*^22^	*U*^33^	*U*^12^	*U*^13^	*U*^23^
I1	0.02772 (10)	0.01391 (8)	0.01487 (8)	0.00169 (6)	0.00351 (6)	0.00146 (6)
O1	0.0302 (12)	0.0232 (10)	0.0257 (10)	−0.0001 (9)	0.0025 (9)	0.0040 (8)
N1	0.0122 (10)	0.0158 (10)	0.0123 (9)	−0.0011 (7)	0.0020 (7)	−0.0026 (7)
N2	0.0141 (10)	0.0104 (9)	0.0141 (9)	0.0002 (7)	0.0011 (7)	0.0004 (7)
N3	0.0157 (10)	0.0151 (10)	0.0089 (9)	0.0000 (7)	−0.0007 (7)	0.0008 (7)
C9	0.0120 (11)	0.0190 (12)	0.0146 (11)	−0.0001 (9)	0.0022 (8)	−0.0020 (9)
C3	0.0063 (10)	0.0146 (11)	0.0167 (11)	−0.0008 (8)	0.0008 (8)	−0.0002 (8)
C14	0.0087 (11)	0.0153 (11)	0.0132 (10)	0.0012 (8)	−0.0001 (8)	−0.0010 (8)
C12	0.0140 (12)	0.0128 (11)	0.0215 (12)	0.0020 (9)	−0.0010 (9)	0.0015 (9)
C5	0.0138 (11)	0.0187 (12)	0.0120 (10)	−0.0018 (9)	−0.0006 (8)	0.0016 (9)
C7	0.0152 (12)	0.0143 (11)	0.0157 (11)	−0.0001 (9)	0.0002 (8)	0.0011 (8)
C11	0.0122 (11)	0.0154 (11)	0.0216 (12)	0.0012 (9)	0.0006 (9)	−0.0033 (9)
C1	0.0117 (11)	0.0144 (11)	0.0157 (11)	−0.0014 (8)	0.0017 (8)	−0.0002 (8)
C13	0.0161 (12)	0.0156 (11)	0.0157 (11)	0.0006 (9)	0.0002 (9)	0.0028 (9)
C4	0.0131 (12)	0.0154 (11)	0.0153 (11)	−0.0007 (9)	0.0000 (8)	0.0013 (8)
C8	0.0157 (12)	0.0197 (12)	0.0133 (11)	0.0010 (9)	0.0010 (9)	0.0005 (9)
C17	0.0164 (13)	0.0222 (13)	0.0250 (13)	−0.0001 (10)	0.0008 (10)	−0.0090 (10)
C15	0.0061 (10)	0.0141 (11)	0.0146 (10)	0.0015 (8)	0.0007 (8)	−0.0004 (8)
C6	0.0091 (11)	0.0144 (11)	0.0143 (10)	−0.0007 (8)	0.0016 (8)	0.0001 (8)
C10	0.0082 (11)	0.0151 (11)	0.0163 (11)	0.0019 (8)	−0.0002 (8)	−0.0013 (9)
C2	0.0140 (11)	0.0171 (11)	0.0122 (10)	0.0003 (9)	0.0019 (8)	0.0012 (8)
C16	0.0161 (12)	0.0172 (12)	0.0155 (11)	−0.0001 (9)	0.0038 (9)	−0.0045 (9)
B1	0.0062 (11)	0.0173 (12)	0.0142 (12)	0.0012 (9)	−0.0006 (9)	0.0004 (10)

### Geometric parameters (Å, º)

**Table:**

O1—H18	0.80 (4)	C5—H4	0.9500
O1—H19	0.77 (4)	C5—C4	1.375 (3)
N1—C5	1.349 (3)	C7—H6	0.9500
N1—C1	1.344 (3)	C7—C8	1.410 (3)
N1—C16	1.485 (3)	C7—C6	1.380 (3)
N2—C6	1.397 (3)	C11—H9	0.9500
N2—B1	1.413 (3)	C11—C10	1.417 (3)
N2—H5	0.83 (3)	C1—H1	0.9500
N3—C14	1.400 (3)	C1—C2	1.383 (3)
N3—B1	1.408 (3)	C13—H11	0.9500
N3—H12	0.81 (3)	C4—H3	0.9500
C9—H8	0.9500	C8—H7	0.9500
C9—C8	1.367 (3)	C17—H16	0.9800
C9—C10	1.418 (3)	C17—H15	0.9800
C3—C4	1.404 (3)	C17—H17	0.9800
C3—C2	1.404 (3)	C17—C16	1.511 (3)
C3—B1	1.581 (3)	C15—C6	1.427 (3)
C14—C13	1.379 (3)	C15—C10	1.424 (3)
C14—C15	1.426 (3)	C2—H2	0.9500
C12—H10	0.9500	C16—H13	0.9900
C12—C11	1.370 (3)	C16—H14	0.9900
C12—C13	1.408 (3)		
			
H18—O1—H19	109 (4)	C14—C13—H11	120.1
C5—N1—C16	119.52 (19)	C12—C13—H11	120.1
C1—N1—C5	120.79 (19)	C3—C4—H3	119.6
C1—N1—C16	119.64 (19)	C5—C4—C3	120.8 (2)
C6—N2—B1	122.7 (2)	C5—C4—H3	119.6
C6—N2—H5	111 (2)	C9—C8—C7	121.1 (2)
B1—N2—H5	127 (2)	C9—C8—H7	119.4
C14—N3—B1	122.82 (19)	C7—C8—H7	119.4
C14—N3—H12	114 (2)	H16—C17—H15	109.5
B1—N3—H12	123 (2)	H16—C17—H17	109.5
C8—C9—H8	119.7	H15—C17—H17	109.5
C8—C9—C10	120.5 (2)	C16—C17—H16	109.5
C10—C9—H8	119.7	C16—C17—H15	109.5
C4—C3—B1	121.5 (2)	C16—C17—H17	109.5
C2—C3—C4	116.6 (2)	C14—C15—C6	121.0 (2)
C2—C3—B1	121.8 (2)	C10—C15—C14	119.6 (2)
N3—C14—C15	117.9 (2)	C10—C15—C6	119.4 (2)
C13—C14—N3	121.9 (2)	N2—C6—C15	117.99 (19)
C13—C14—C15	120.2 (2)	C7—C6—N2	122.0 (2)
C11—C12—H10	119.4	C7—C6—C15	120.0 (2)
C11—C12—C13	121.3 (2)	C9—C10—C15	118.8 (2)
C13—C12—H10	119.4	C11—C10—C9	122.7 (2)
N1—C5—H4	119.7	C11—C10—C15	118.4 (2)
N1—C5—C4	120.6 (2)	C3—C2—H2	119.7
C4—C5—H4	119.7	C1—C2—C3	120.5 (2)
C8—C7—H6	120.0	C1—C2—H2	119.7
C6—C7—H6	120.0	N1—C16—C17	113.04 (19)
C6—C7—C8	120.1 (2)	N1—C16—H13	109.0
C12—C11—H9	119.6	N1—C16—H14	109.0
C12—C11—C10	120.7 (2)	C17—C16—H13	109.0
C10—C11—H9	119.6	C17—C16—H14	109.0
N1—C1—H1	119.7	H13—C16—H14	107.8
N1—C1—C2	120.6 (2)	N2—B1—C3	121.8 (2)
C2—C1—H1	119.7	N3—B1—N2	117.4 (2)
C14—C13—C12	119.7 (2)	N3—B1—C3	120.8 (2)
			
N1—C5—C4—C3	0.0 (3)	C8—C9—C10—C11	−178.3 (2)
N1—C1—C2—C3	−0.1 (3)	C8—C9—C10—C15	−0.3 (3)
N3—C14—C13—C12	−179.6 (2)	C8—C7—C6—N2	178.6 (2)
N3—C14—C15—C6	2.0 (3)	C8—C7—C6—C15	−1.0 (3)
N3—C14—C15—C10	−178.65 (19)	C15—C14—C13—C12	−0.4 (3)
C14—N3—B1—N2	1.2 (3)	C6—N2—B1—N3	2.3 (3)
C14—N3—B1—C3	−177.6 (2)	C6—N2—B1—C3	−178.9 (2)
C14—C15—C6—N2	1.2 (3)	C6—C7—C8—C9	−0.1 (3)
C14—C15—C6—C7	−179.2 (2)	C6—C15—C10—C9	−0.7 (3)
C14—C15—C10—C9	179.9 (2)	C6—C15—C10—C11	177.3 (2)
C14—C15—C10—C11	−2.1 (3)	C10—C9—C8—C7	0.8 (4)
C12—C11—C10—C9	178.5 (2)	C10—C15—C6—N2	−178.15 (19)
C12—C11—C10—C15	0.5 (3)	C10—C15—C6—C7	1.4 (3)
C5—N1—C1—C2	−2.8 (3)	C2—C3—C4—C5	−2.6 (3)
C5—N1—C16—C17	−96.2 (2)	C2—C3—B1—N2	−0.9 (3)
C11—C12—C13—C14	−1.3 (4)	C2—C3—B1—N3	177.9 (2)
C1—N1—C5—C4	2.8 (3)	C16—N1—C5—C4	−174.6 (2)
C1—N1—C16—C17	86.3 (3)	C16—N1—C1—C2	174.7 (2)
C13—C14—C15—C6	−177.3 (2)	B1—N2—C6—C7	177.0 (2)
C13—C14—C15—C10	2.1 (3)	B1—N2—C6—C15	−3.4 (3)
C13—C12—C11—C10	1.2 (4)	B1—N3—C14—C13	176.0 (2)
C4—C3—C2—C1	2.7 (3)	B1—N3—C14—C15	−3.2 (3)
C4—C3—B1—N2	−179.5 (2)	B1—C3—C4—C5	176.1 (2)
C4—C3—B1—N3	−0.8 (3)	B1—C3—C2—C1	−176.0 (2)

### Hydrogen-bond geometry (Å, º)

**Table:**

*D*—H···*A*	*D*—H	H···*A*	*D*···*A*	*D*—H···*A*
O1—H18···I1^i^	0.80 (4)	2.87 (4)	3.643 (3)	162 (4)
O1—H19···I1^ii^	0.77 (4)	3.04 (4)	3.793 (3)	167 (4)
N2—H5···I1	0.83 (3)	2.95 (3)	3.764 (2)	171 (2)
N3—H12···O1	0.82 (3)	2.23 (3)	3.046 (3)	172 (3)
C2—H2···I1	0.95	3.14	4.081 (2)	171
C16—H14···I1^iii^	0.99	2.98	3.840 (3)	145
C16—H13···I1^iv^	0.99	3.15	3.946 (3)	138

Symmetry codes: (i) −*x*+2, −*y*+2, −*z*+1; (ii) −*x*+1, −*y*+2, −*z*+1; (iii) *x*, *y*, *z*−1; (iv) *x*, −*y*+3/2, *z*−1/2.
